# Highly hydrophilic poly(vinylidene fluoride)/meso-titania hybrid mesoporous membrane for photocatalytic membrane reactor in water

**DOI:** 10.1038/srep19148

**Published:** 2016-01-12

**Authors:** Meng Wang, Guang Yang, Peng Jin, Hao Tang, Huanhuan Wang, Yong Chen

**Affiliations:** 1 School of Chemical and Environmental Engineering, Shanghai Institute of Technology, Shanghai 201418, China

## Abstract

The high hydrophobicity of poly(vinylidene fluoride) (PVDF) membrane remains an obstacle to be applied in some purification processes of water or wastewater. Herein, a highly hydrophilic hybrid mesoporous titania membrane composed of mesoporous anatase titania (meso-TiO_2_) materials inside the three-dimensional (3D) macropores of PVDF membrane was successfully prepared by using the dual-templated synthesis method combined with solvent extraction and applied as the photocatalytic membrane reactor for the photodegredation of organic dye in water. The structure and the properties of as-prepared hybrid membranes were characterized by scanning electron microscopy (SEM), energy dispersive X-ray spectroscopy (EDS), transmission electron microscopy (TEM), X-ray diffraction (XRD), nitrogen adsorption–desorption and contact angle measurements. It was found that the hydrophilicity of PVDF membrane can be significantly improved by filling mesoporous TiO_2_ inside the 3D macropores of PVDF membrane. Moreover, such a PVDF/meso-TiO_2_ hybrid membrane exhibits promising photocatalytic degradation of dye in water due to the existence of mesoporous anatase TiO_2_ materials inside PVDF membrane. This study provides a new strategy to simultaneously introduce hydrophilicity and some desirable properties into PVDF and other hydrophobic membranes.

During the past decade, lots of novel hybrid mesoporous membranes composed of mesoporous materials, such as meso-silica or meso-titania, not on the surface but within the inside-pores of some porous membranes have attracted great attention due to their unique structure with pores-in-pores^1^,^2^,^3^,^4^, and widely potential applications in the templated-syntheses of nanomaterials^5^,^6^, nanofiltration^7^,^8^,^9^,^10^, sensors^11^,^12^,^13^, electronic devices^14^ and lithium batteries^15^. Normally, dual templates including various porous membranes and surfactants have been respectively employed as the morphology-directing hard template and the structure-directing soft template in the synthesis of hybrid mesoporous membranes. So far, all of those reports on hybrid mesoporous membranes have mainly focused on two kinds of commercial filtration membranes with one-dimensional cylindrical channels, namely porous anodic alumina (PAA)^1^,^2^,^3^,^4^,^5^,^6^,^7^,^16^ and polycarbonate (PC) membranes^8^,^11^,^17^, both of which were applied as the hard templates. Recently, other commercial porous membrane with different structure and property, such as cellulose and polyethylene terephthalate membranes, have also been used as the new hard templates for the fabrication of hybrid mesoporous membrane by Yamauchi *et al.*^9^ and our group^10^,^12^. However, it should be noted that all the hard templates mentioned above are hydrophilic membranes. To date, those hydrophobic porous membranes have rarely been applied as the hard templates to fabricate such hybrid mesoporous membranes, for example, poly(vinylidene fluoride) (PVDF) membranes.

PVDF membranes have been considered as one of the more popular membrane materials widely applied in the membrane separation processes because of their high mechanical strength, thermal stability and excellent resistance to corrosion from many chemicals and organic solvents^18^,^19^,^20^. Nevertheless, the high hydrophobicity and low surface energy of PVDF membranes has become a great drawback for their applications in some separation and purification processes of water and wastewater^19^,^20^,^21^,^22^,^23^,^24^. Therefore, it is very significant to improve the hydrophilicity of PVDF membranes through versatile methods mainly including surface modification and blending modification^19^,^20^. Surface modification is achieved by coating or grafting a functional layer on the prepared membrane surface, but not the pores inside the membrane^21^,^22^, which is different from the blending modification based on a single step^23^,^24^,^25^. Since the surfaces and the inside-pores of PVDF membranes can provide a window of opportunity to be modified^19^,^20^ and those hybrid mesoporous membranes prepared by using dual-templated synthesis method can introduce desirable properties into the original hydrophilic membranes^1^,^2^,^3^,^4^,^5^,^6^,^7^,^8^,^9^,^10^,^11^,^12^,^13^,^14^,^15^,^16^,^17^, it is necessary to further explore the fabrication of hybrid mesoporous membranes by employing hydrophobic porous membranes as the hard templates.

In this study, we report a new hybrid mesoporous titania membrane composed of mesostructured anatase TiO_2_ materials inside 3D macropores of PVDF membrane, which was prepared by using a commercially available PVDF membrane and poly (ethylene oxide)-block-poly (propylene oxide)—block-poly (ethylene oxide) (Pluronic P123) as the hard and the soft templates, respectively. In addition, solvent extraction instead of calcination was used to remove surfactant P123 from the TiO_2_ mesopores inside PVDF membrane in order to prevent the destruction of PVDF under high temperature and keep the structural integrity of hybrid mesoporous membranes based on our previous work^8^,^10^,^11^,^12^. It was found that the formation of meso-TiO_2_ in PVDF not only effectively improved the hydrophilicity of PVDF membrane, but also incorporated attractive functions, such as adsorption and photocatalysis, into original PVDF membrane. Moreover, the results of comparative experiment performed by using different hybrid membranes with or without solvent extraction (respectively abbreviated as PVDF/meso-TiO_2_ and PVDF/meso-TiO_2_/P123) revealed that the main properties of hybrid mesoporous titania membrane, including hydrophilicity and photocatalysis, were affected by the surfactant P123 present inside the TiO_2_ mesopores. This study focusing on highly hydrophobic PVDF membrane was expected to be of profound significance because it not only enriched the dual-templated synthesis of hybrid mesoporous membranes, but also offered a novel strategy to improve the properties of hydrophobic membranes.

## Results

**Figure 1:**
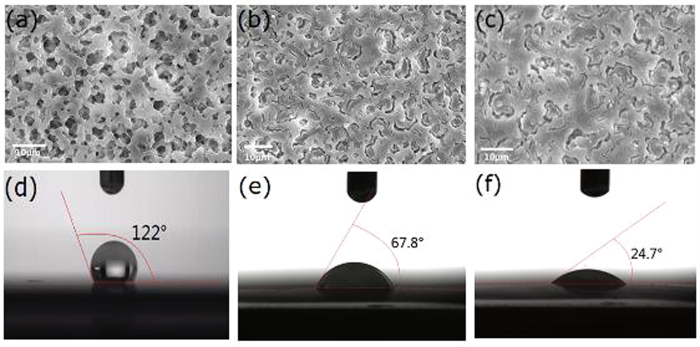
SEM images of the top-view of (**a**) commercial PVDF, (**b**) PVDF/meso-TiO_2_ and (**c**) PVDF/meso-TiO_2_/P123 membranes. Images of water droplet on the external surface of (**d**) commercial PVDF, (**e**) PVDF/meso-TiO_2_ and (**f**) PVDF/meso-TiO_2_/P123 membranes.

**Figure 2:**
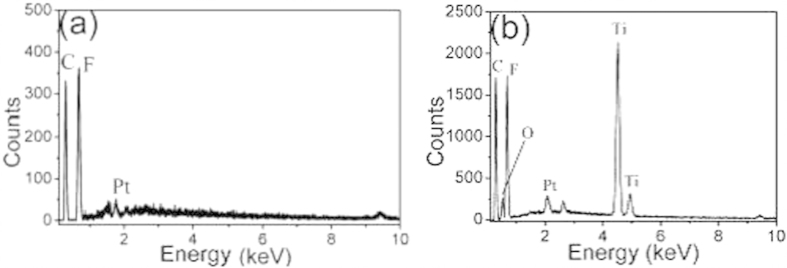
EDS data of (**a**) commercial PVDF and (**b**) PVDF/meso-TiO_2_ membranes.

Figure 1a–c show the SEM images of the top-view of PVDF, PVDF/meso-TiO_2_, and PVDF/meso-TiO_2_/P123 membranes, respectively. Figures 1a and S1 clearly demonstrate that the commercially available PVDF membrane consists of 3D macroporous texture. Comparing Fig. 1b,c with Fig. 1a, it is found that 3D macroporous pores of PVDF are completely filled with some rod-like materials after the dual-templated synthesis performed with or without solvent extraction. When PVDF present in PVDF/meso-TiO_2_ membrane was completely etched by calcination under high temperature, a number of free layers composed of rods could be obtained (Fig. S2), which almost duplicate the 3D macroporous structure of PVDF membrane. The elemental compositions of the above mentioned materials were tested by energy dispersive X-ray spectroscopy (EDS) analyses. Along with the component elements (F and C) of PVDF (Fig. 2a), titanium (Ti) and oxygen (O) can be detected on the surface of PVDF/meso-TiO_2_ (Fig. 2b). Also, Ti and O can be detected from the free layers composed of rod-like materials released from PVDF membrane (Fig. S3a), indicating that the materials formed inside 3D macroporous pores of PVDF membrane should be titania, which is further verified by the results of their corresponding EDS mappings (Fig. S3b,c).

Contact angle measurements were widely used for the characterization of the hydrophobicity or hydrophilicity of membrane surfaces^22^,^23^,^25^. The corresponding water contact angles of PVDF, PVDF/meso-TiO_2_ and PVDF/meso-TiO_2_/P123 are shown in Fig. 1d–f, which clearly demonstrate that the original hydrophobicity of PVDF membrane changes to high hydrophilicity when the 3D macroporous pores of PVDF are filled with TiO_2_ although the difference among the macroscopic images of three membranes is not obvious (Fig. S4). The pristine PVDF membrane has the highest initial contact angle of 122° from Fig. 1d, which indicates the intrinsic and superior hydrophobicity of PVDF membrane due to the intensive electronegative characteristics of fluorine element resulting in the low surface energy of fluoropolymers and weak affinity of PVDF toward water^19^,^20^. After the dual-templated synthesis with or without solvent extraction, the initial contact angle of PVDF membrane remarkably decreases in the contact angle from 122° to 67.8° (PVDF/meso-TiO_2_, Fig. 1e) and 24.7° (PVDF/meso-TiO_2_/P123, Fig. 1f). This result illustrates that the hydrophilicity of PVDF membrane can be significantly improved by the incorporation of TiO_2_ into the macropores of PVDF membrane, which should be ascribed to the introduction of hydrophilic group of TiO_2_, hydroxyl^26^, to the original hydrophobic PVDF membrane. Meanwhile, the smaller water contact angle of PVDF/meso-TiO_2_/P123 indicates that the surfactant, amphiphilic triblock polymer P123, is also helpful to lower the surface energy of PVDF membrane owing to its amphiphilic groups^27^. The hydration effect between those hydrophilic groups and water should play the key role in the improvement of hydrophilicity of PVDF membrane.

**Figure 3:**
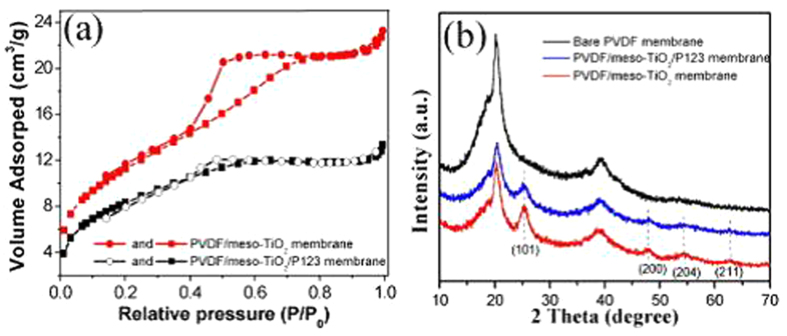
(**a**) Nitrogen adsorption–desorption isotherm plots of PVDF/meso-TiO_2_ and PVDF/meso-TiO_2_/P123 membranes. (**b**) XRD patterns of commercial PVDF membrane, as-prepared PVDF/meso-TiO_2_/P123 and PVDF/meso-TiO_2_ membranes.

Figure 3a shows the nitrogen adsorption–desorption isotherm curves of PVDF/meso-TiO_2_ and PVDF/meso-TiO_2_/P123 membranes, both of which exhibit the typical type-IV mesoporous adsorption–desorption behaviors with a hysteresis loop ascribed to the capillary condensation^15^,^28^,^29^. Additionally, the inflection positions of those two curves in P/P_o_ are almost similar to those in previous reports on the mesoporous TiO_2_ materials by using P123 as the structure-directing soft template^15^. The average pore radius and the Brunauer–Emmett–Teller (BET) specific surface area (*S*_BET_) of as-prepared hybrid mesoporous membranes increased from 2.7 nm and 30.7 m^2^ g^−1^ for PVDF/meso-TiO_2_/P123 to 3.5 nm and 41.5 m^2^ g^−1^ for PVDF/meso-TiO_2_, reflecting the efficiency of solvent extraction for the removal of surfactants from as-prepared hybrid mesoporous membranes as reported previously^8^,^10^,^11^,^12^. Figure 3b shows the corresponding wide-angle X-ray diffraction (WAXRD) patterns of PVDF, PVDF/meso-TiO_2_/P123, and PVDF/meso-TiO_2_ membranes. The XRD pattern corresponding to such a commercial PVDF membrane exhibits two main diffraction peaks at 2θ = 20.1°, 36.4°, and 39.7°, which is in good agreement with the characteristic peaks of PVDF crystals reported previously^30^. For PVDF/meso-TiO_2_/P123 and PVDF/meso-TiO_2_ membranes, in addition to the diffraction peaks of PVDF, four new diffraction peaks located at 25.0°, 48.1°, 54.9°, and 62.5° (2θ) are observed in their corresponding XRD patterns, which can be respectively assigned to (101), (200), (211), and (204) diffraction planes of the anatase phase of TiO_2_ (JCPDS No. 84–1286)^15^, indicating that the mesostructured TiO_2_ formed in PVDF is predominantly composed of anatase TiO_2_ crystallites. Notably, all the diffraction peaks corresponding to anatase TiO_2_ crystallites become more intense after the removal of P123 from hybrid membrane by the comparison of curve E to curve D, which further confirms the efficiency of solvent extraction for removing the surfactants from hybrid mesoporous membranes as discussed above.

**Figure 4:**
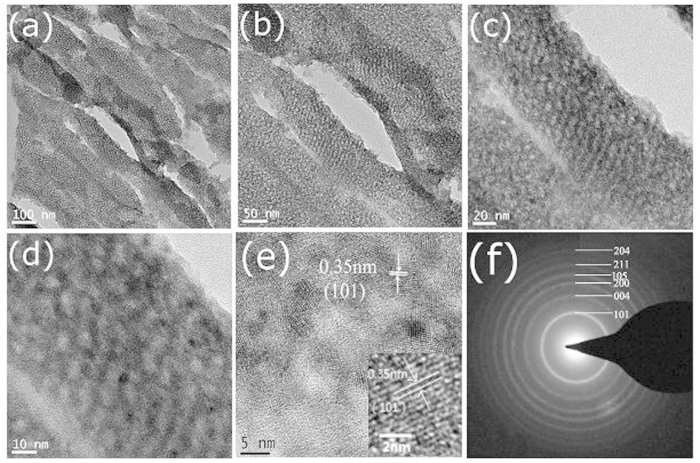
TEM images with different magnifications (**a–e**) of the mesostructured TiO_2_ materials after PVDF/meso-TiO_2_ membranes were embedded in an epoxy resin and cut by mechanical polishing for measurements. (**f**) SAED pattern recorded on the sample.

The mesostructure and crystalline structure of TiO_2_ formed in PVDF were further investigated by transmission electron microscopy. Some typical TEM images of PVDF/meso-TiO_2_ are shown in Fig. 4a–e. Figure 4a–d clearly show that the layers composed of mesoporous rods have a mesoporous structure with a wormhole-like nanochannel network, which is similar to those hybrid mesoporous membranes composed of titania fibers inside other porous organic membrane^31^,^32^. According to the high-resolution TEM (Fig. 4d,e), the average values of mesopore radius and crystal size in the layers are respectively estimated to be about 3.0 nm and 5–7 nm, which are close to the values reported previously^15^. In addition, the characteristic anatase lattice fringes of TiO_2_ materials can obviously be seen and the distance of aligned lattice fringe spacing is evaluated to be 0.35 nm from the inset of Fig. 4e, which is consistent with the d101 spacing of anatase TiO_2_^33^. Moreover, the anatase phase of above mentioned mesoporous TiO_2_ is further confirmed by analyzing the selected area electron diffraction (SAED) pattern (Fig. 4f), which presents several strong Debye–Scherrer rings corresponding to the reflections of TiO_2_ anatase phase^34^,^35^. Among the common crystalline forms of TiO_2_, anatase is, in general, recognized to be the most active phase excited by ultraviolet (UV) irradiation^35^,^36^,^37^,^38^.

Since the hydrophilicity of porous PVDF membrane can be efficiently improved by filling its pores with mesoporous TiO_2_ composed of anatase crystalline phase as discussed above. Another objective of this study was to investigate the photocatalytic property of as-prepared hybrid mesoporous TiO_2_ membranes. Herein, methyl orange (MO), a typical dye pollutant in the textile industry extensively studied to evaluate the photocatalytic activity of various anatase TiO_2_ materials^36^,^37^,^38^, was selected as a model compound to investigate the photocatalytic activity of as-prepared hybrid mesoporous TiO_2_ membranes. According to our previous work^11^, hybrid mesoporous membranes exhibit adsorption property to some extent. Therefore, it is necessary to examine the adsorption property of hybrid mesoporous TiO_2_ membranes prior to the experiments on their photocatalytic property.

**Figure 5:**
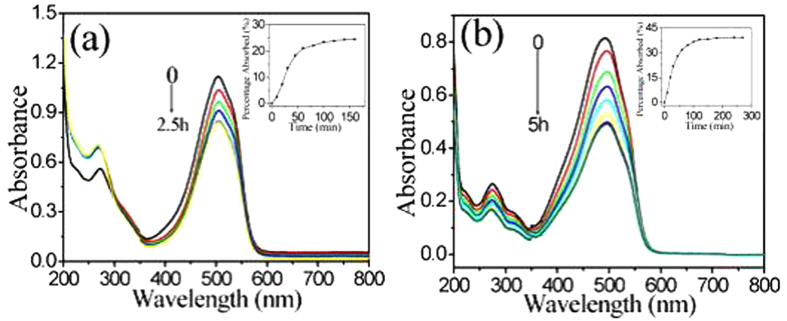
The change of UV–Vis absorption spectra of MO solution adsorbed by (**a**) PVDF/meso-TiO_2_/P123 and (**b**) PVDF/meso-TiO_2_ membranes under the dark test. Inset: adsorption rate curves for MO.

Figure 5 shows the absorption spectra of MO in the presence of a piece of PVDF/meso-TiO_2_ or PVDF/meso-TiO_2_/P123 membrane performed under the dark test. It is found that the adsorption reaches equilibrium after 100 min and the maximum adsorption percentage for MO on PVDF/meso-TiO_2_/P123 is 24% (Fig. 5a). As far as PVDF/meso-TiO_2_ is concerned, Fig. 5b shows that the time to achieve adsorption equilibrium and the maximum adsorption percentage increase to 120 min and 39%, respectively. The difference of maximum adsorption percentage between PVDF/meso-TiO_2_ and PVDF/meso-TiO_2_/P123 membranes should be attributed to the increase in *S*_BET_ of PVDF/meso-TiO_2_ membrane because of the removal of P123 from hybrid membranes by solvent extraction as discussed above. The adsorption capacity of PVDF/meso-TiO_2_ membrane for MO was estimated about 4.0 mg g^−1^, which is much larger than that of hybrid mesoporous silica membrane as reported previously^11^. The reason for the larger adsoprtion capacity of PVDF/meso-TiO_2_ can be mainly due to the higher *S*_BET_ of PVDF/meso-TiO_2_ than those hybrid mesoporous membranes prepared by employing porous membranes with 1D channels^8^,^10^,^11^,^12^, which implies that it should help to improve the adsorption property of hybrid mesoporous membranes by using porous membranes with 3D pore structure as the hard templates.

**Figure 6:**
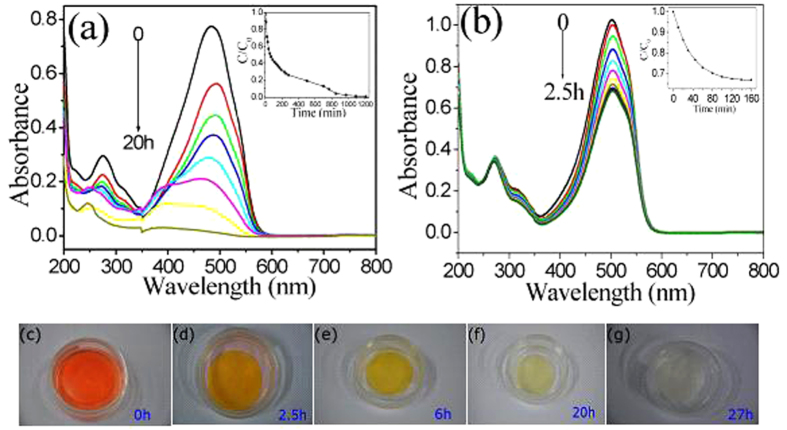
The change of UV–Vis absorption spectra of MO solution degraded by (**a**) PVDF/meso-TiO_2_ and (**b**) PVDF/meso-TiO_2_/P123 membranes. Insets in (**a,b**) degradation rates of MO under UV irradiation using different hybrid membranes. (**c–g**) Images of photocatalytic degradation of MO in the presence of PVDF/meso-TiO_2_ membrane during different times.

The photocatalytic activity of the PVDF/meso-TiO_2_ membrane was evaluated by degradation of MO under UV irradiation according to the previous reports^35^,^36^. It is found that the absorption band intensity of MO decreases negligibly in the presence of PVDF membrane, which just floats on the aqueous solution and remains colorless without any adsorption of MO by the PVDF membrane (Fig. S5), reflecting the high hydrophobicity of original PVDF as shown as Fig. 1d and the extreme stability of MO molecules under normal conditions^35^. When a piece of PVDF/meso-TiO_2_ membrane was put into the same MO solution, it quickly wets and sinks into the MO aqueous solution (Fig. 6c–g), which further demonstrates the high hydrophilicity of hybrid membrane after the modification of PVDF by meso-TiO_2_. Moreover, as shown in Fig. 6a and the corresponding images (Fig. 6c–g), the absorption band intensity and the corresponding color depth of aqueous solution of MO rapidly decease with the increase in irradiation time within 300 min, and nearly disappear after 20 h. Moreover, the PVDF/meso-TiO_2_ membrane immersed in water also change to colorless after 27 h. As a comparison, Fig. 6b shows the photoactivity of PVDF/meso-TiO_2_/P123 membrane. It is found that the absorption band intensity of aqueous solution of MO deceases slowly with the increase of irradiation time, and remains almost unchanged after 120 min and even one day, which indicates that it is impossible for PVDF/meso-TiO_2_/P123 membrane to completely photodegrade MO. The time profiles of *C*_t_/*C*_0_ under UV irradiation (the insets in Fig. 6a,b) further demonstrate that the decrease in percentage of MO in the presence of PVDF/meso-TiO_2_ membrane within 60 min is about 50%, which can reach 70% after 300 min. However, under the same UV irradiation, the maximun percentage decrease of MO using PVDF/meso-TiO_2_/P123 membrane was 33%, which was only 9% greater than the maximum adsorption percentage obtained by using the same membrane under dark test and much smaller than that obtained by employing PVDF/meso-TiO_2_ membrane. All the above mentioned results indicated that PVDF/meso-TiO_2_ membrane exhibited better photodegradation ability toward MO than PVDF/meso-TiO_2_/P123 membrane.

**Figure 7:**
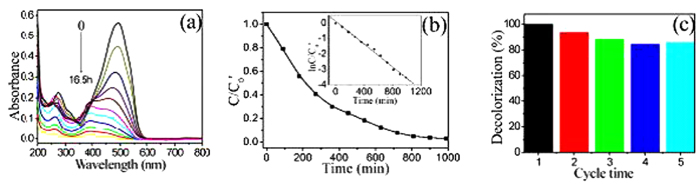
(**a**) The change of UV–Vis absorption spectra of MO solution and (**b**) the degradation rates of MO by using PVDF/meso-TiO_2_ as the photocatalytic membrane reactor under UV irradiation; Inset in (**b**): the ln(C/C_0_’) *vs*. time curve of photodegradation of MO. (**c**) The reuse activity of PVDF/meso-TiO_2_ for photodegradation of MO.

Figure 7a,b show the absorption spectra and the time profiles of *C*/*C*_0_’ (*C*_0_’ is the concentration of MO after the adsorption/desorption equilibrium) of MO under UV irradiation after immersing a piece of PVDF/meso-TiO_2_ membrane into the aqueous solution of MO for 300 min under the dark environment until adsorption/desorption equilibrium is achieved. It is found from those two figures that the absorption band intensity and the corresponding *C*/*C*_0_’ of MO in the presence of PVDF/meso-TiO_2_ membrane decrease about 70% within 270 min, which can reach 90% after 720 min. Moreover, the experimental datum can be fitted well by using the pseudo first-order kinetic equation ln(*C*_t_/*C*_0_’) = −*kt* (the inset of Fig. 7b) and the reaction rate constant (*k*) of MO photodegradation is calculated to be 3.7 × 10^−3^ min^−1^ for PVDF/meso-TiO_2_ membrane, which is larger than the value determined for Degussa P25 well-known to have good photocatalytic activity^36^. It should be pointed out that the intensity of UV light source employed here is only 25 W, which is a little higher than the value (15 W) reported previously^36^ but much lower than that by using UV light source with 300 W^37^. The main reason for the better activity of PVDF/meso-TiO_2_ membrane should be ascribed to their larger *S*_BET_, which offers more active sites and allows more reactants to absorbed on the surface of the photocatalyst^36^,^39^,^40^. Additionally, Fig. 7c shows that the photocatalysis efficiency of PVDF/meso-TiO_2_ remains constant after three consecutive cycles and exhibits a little change (<15%) after five recycles, which demonstrates that the meso-TiO_2_ formed in PVDF membrane presents reasonable stability under UV light irradiation. Thus, it can be inferred that as-prepared PVDF/meso-TiO_2_ membrane can be applied as the photocatalytic membrane reactor with good activity and stability toward the photocatalytic degradation of MO dye. Furthermore, the results of photodegradation of MO conducted under different concentration of MO (C_MO_) show that the time for the complete degradation of MO by PVDF/meso-TiO_2_ membrane also depends on the C_MO_ (Fig. S6), which changes from 16.5 h (10 mg/L MO) to 4.5 h (5 mg/L MO) and 2.5 h (1 mg/L MO).

## Discussion

In this study, a relatively simple and effective route was developed to introduce both hydrophilic character and photocatalytic property into hydrophobic PVDF membrane via filling of mesoporous anatase TiO_2_ inside the 3D macropores of PVDF membrane by dual-templated synthesis method combined with solvent extraction. It is found that as-prepared PVDF/meso-TiO_2_ hybrid mesoporous membrane exhibits high hydrophilicity and photocatalytic efficiency toward degradation of MO. Obviously, such a hydrophilic and photocatalytic membrane offered advantage of easy recyclability because it can be not only conveniently removed from the reaction system without any secondary pollution, but also reused and recycled without obvious loss in the catalytic efficiency, which undoubtedly overcomes one significant drawback of using powdered TiO_2_ photocatalysts^28^,^34^,^35^.

**Figure 8:**
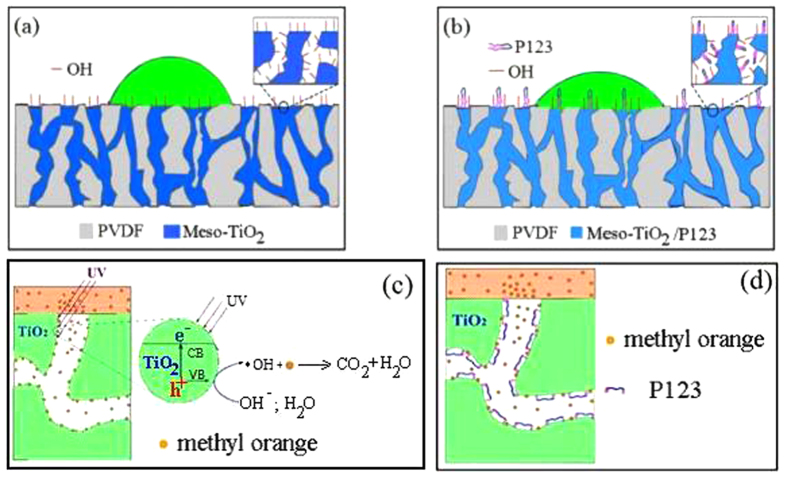
Schemes for the mechanism of the hydrophilicity of (**a**) PVDF/meso-TiO_2_ and (**b**) PVDF/meso-TiO_2_/P123 membranes, as well as the difference between the photocatalytic abilities of (**c**) PVDF/meso-TiO_2_ and (**d**) PVDF/meso-TiO_2_/P123 membranes.

Based on all above results and discussion, the mechanism of such a photocatalytic membrane reactor for the photodegradation of MO in water by filling meso-TiO_2_ in PVDF membrane can be illustrated as Fig. 8a–d, which should be mainly related to the three properties of as-prepared membranes including hydrophilicity, adsorption and photocatalysis. On the one hand, the results of contact angle measurements (Fig. 1d–f) clearly illuminate that the hydrophilic groups derived from mesoporous materials with or without solvent extraction formed inside PVDF can lower the surface energy of PVDF membrane and improve its hydrophilicity, as shown as Fig. 8a for PVDF/meso-TiO_2_ membrane and Fig. 8b for PVDF/meso-TiO_2_/P123 membrane. Besides the improvement of hydrophilicity, the formation of mesopores of TiO_2_ in the pores of PVDF (pores-in-pores) can allow more MO to be absorbed onto and into the membrane because of the enlargement of specific surface area, which should also play an important role in the whole process of photodegradation of MO^36^,^39^. On the other hand, the complete photocatalytic discolorization of MO by PVDF/meso-TiO_2_ membrane (Fig. 6a,g) indicates that it is possible for meso-TiO_2_ in PVDF to oxidize organic pollutants into non-toxic materials after the adsorption of MO onto the surface of meso-TiO_2_(Fig. 8c), which should be induced by the photogenerated holes and some radicals^37^,^39^,^41^,^42^,^43^,^44^,^45^,^46^, especially very reactive hydroxyl radicals (·OH) formed by the reactions of the holes left in the valence band (VB) of TiO_2_ with the adsorbed water or surface hydroxyl and considered as the major active species responsible for the photocatalytic oxidation^42^,^43^,^44^,^45^,^46^. As a comparison, the low efficiency of PVDF/meso-TiO_2_/P123 membrane for the photodegradation of MO (Fig. 6b) indicates that the existence of surfactant P123 in TiO_2_ mesopores should hinder the desirable photoactivity of such hybrid mesoporous membranes mainly due to the unexpected coverage of photocatalytic active sites in the mesopores by surfactant P123 (Fig. 8d), though it indeed helps to enhance the hydrophilicity of PVDF membrane.

Due to the the high reactivity and very short lifetime of ·OH, it is very difficult to detect such a reactive radical^46^. Therefore, some indirect detection methods have been developed to investigate ·OH produced by various phtocatalysts during the past several decades, such as photoluminescence (PL) technique^45^,^46^. In this work, PL technique was also applied to detect the ·OH produced by the mesoporous anatase TiO_2_ inside the 3D macropores of PVDF membrane according to the previous reports^45^,^46^ by using coumarin (COU) as the test molecule, which can react with ·OH to form the highly fluorescent compound 7-hydroxycoumarin (7HC). However, no obvious PL signal of 7HC could be observed after the 10^−3^ mol L^−1^ COU solution containing a piece of PVDF/meso-TiO_2_ membrane was irradiated by UV light (Fig. S7A). Considering that the photogenerated ·OH by photocatalyst presents two kinds of forms, namely free ·OH in solution and surface-bound ·OH on sample surface, the possible reason for the rare PL signal of 7HC observed herein could be ascribed to two aspects. One is that the trapping efficiency of ·OH formed on TiO_2_ photocatalyst is only 4.7% by PL technique using COU as probe molecule^47^, and such a value obtained herein may be lower due to the unique pores-in-pores structure of PVDF/meso-TiO_2_ membrane, which is distinct from those powdered TiO_2_ materials. The other should be owing to the possible adsorption of most of 7HC into the PVDF/meso-TiO_2_ membrane. Indeed, the adsorption experiments showed that the PL intensity of standard compound 7HC (10 μmol L^−1^) in a 10^−3^ mol L^−1^ COU aqueous solution decreased quickly with the time especially during the initial period (15 min) (Fig. S7B). Therefore, it can be assumed that most of ·OH produced by the PVDF/meso-TiO_2_ membrane should be the surface-bound ·OH formed on the inner surface of meso-TiO_2_ in such a hybrid membrane, as well as the compounds 7HC based on the reactions between surface-bound ·OH or free ·OH and COU should mainly be formed inside the TiO_2_ mesopores of PVDF/meso-TiO_2_ membrane, which can be quickly adsorbed onto the inner surface of TiO_2_ mesopores (Fig. S7B) leading to the rare PL signal 7HC observed herein (Fig. S7A). Based on this assumption, it is better to understand the obvious difference of photodegradation ability toward MO between PVDF/meso-TiO_2_ and PVDF/meso-TiO_2_/P123 membranes. Owing to the existence of P123 inside PVDF/meso-TiO_2_/P123 membrane, the amount of surface hydroxyl on the mesopores inside PVDF/meso-TiO_2_/P123 membrane should be lower than that in PVDF/meso-TiO_2_ membrane, which leads to much less surface-bound ·OH formed in PVDF/meso-TiO_2_/P123 than PVDF/meso-TiO_2_ to attack the adsorbed pollutant molecules to produce oxidized species and/or decomposed products, as shown as Fig. 8c,d.

As a result, PVDF/meso-TiO_2_/P123 mainly presents the properties of hydrophilicity and adsorption. Only the PVDF/meso-TiO_2_ membrane prepared by the combination of dual-templated synthesis with solvent extraction can simultaneously introduce the desired properties of hydrophilicity and photocatalysis into hydrophobic PVDF membrane, both of which are essential for its potential applications as the photocatalytic membrane reactor in the waste water treatment. This study provides a general methodology for the preparation of more versatile hybrid mesoporous membranes by employing hydrophobic membranes as the hard templates. From a more practical application viewpoint, it is still necessary to fabricate hybrid mesoporous titania membranes containing metal/nonmetal-doped heterojunctions in the future for their better photocatalytic performance especially under visible light based on the improvement of the generation and separation of photoinduced electron-hole pairs in titania^39^,^40^,^41^,^43^,^48^,^49^. In addition, the structure-regulation of TiO_2_ to form the hierarchical macro/mesoporous composites^42^,^43^,^44^,^50^,^51^,^52^ inside PVDF membrane is expected to be helpful for the further enhancement of photocatalytic activity of such a photocatalytic membrane reactor.

## Methods

### Materials

PVDF membranes (Ф ~ 47 mm) with thickness of 20 μm and pore diameters of 0.22 μm were obtained from Shanghai Mosu Science Equipment Co., Ltd., China. The Pluronic P123 triblock copolymer denoted as EO_20_PO_70_EO_20_ (molar weight ~5800 g mol^−1^) was purchased from Sigma Aldrich. Titanium tetraisopropoxide (TTIP), methyl orange (MO), ethanol, aqueous solution of HCl (12 mol L^−1^) and other chemicals were obtained from Sinopharm Chemical Reagent Co., Ltd. All chemical reagents were of analytical grade and used as received without further purification. Distilled water was used for the preparation of all the solution employed in this study.

### Dual-templated synthesis of PVDF/meso-TiO_2_ hybrid mesoporous membrane

TTIP was used as the titania source and the triblock copolymer P123 was employed as the structure-directing agent (soft template). First, a clear sol with a mass composition of 4.9 TTIP: 2 P123: 4.1 HCl: 13.9 EtOH was obtained by stirring at room temperature for 1 h^15^. The sol was further diluted by absolute ethanol (3:1 v/v of ethanol to the initial sol). After that, as shown in Fig. S8, a PVDF membrane was submerged into as-prepared sol (6 mL) in a small open vessel with an inner diameter of about 5.5 cm. Subsequently, the open vessel containing the PVDF membrane and precursor solution was sealed inside another larger vessel to construct the entire reaction unit and aged at 60 °C for one day. Later, the obtained product was ultrasonically washed three times with deionized water and alcohol, and then dried at 80 °C to obtain hybrid mesoporous TiO_2_ membrane containing P123 (PVDF/meso-TiO_2_/P123). Finally, the surfactants P123 were removed from PVDF/meso-TiO_2_/P123 membrane by solvent-extraction using the mixed solvents containing ethanol and water (molar ratio 1:1)^11^ to afford PVDF/meso-TiO_2_ hybrid mesoporous membrane.

### Characterization

SEM images were obtained on a HITACHI-3400 equipped with Quantax-400 EDX (Bruker. Ins. Germany). As-prepared PVDF/meso-TiO_2_ hybrid mesoporous membranes were embedded in an epoxy resin and cut by mechanical polishing for the TEM measurements. High resolution transmission electron microscopy (HRTEM) images were collected using Tecnai G^2^ F20 S-Twin (FEI Co. USA) microscope operated at 200 kV. WAXRD patterns were recorded on PW3040/60 X’Pert PRO X-ray (Panalytical. Ins. Netherlands). Nitrogen adsorption–desorption isotherms were obtained by a Tristar II 3020 analyzer (Micromeritics, USA). BJH methods were used to estimate the pore size. The contact angle between water and the external surface of membrane was measured to evaluate the membrane hydrophobicity using a JC2000D1 contact angle meter (Shanghai Zhongchen Digital Technic Apparatus Co., Ltd, China).

### Measurements of adsorption and photocatalytic activity

In a characteristic adsorption experiment, the adsorption activity measurement of PVDF/meso-TiO_2_ or PVDF/meso-TiO_2_/P123 membranes was performed at ambient temperature in a 0.13 L sealed plastic container by the adsorption of MO (20 mL) with an initial concentration of 10 mg L^−1^. The process of adsorption was performed in the absence of light (dark experiment). The apparatus employed for the photocatalytic degradation of MO consisted of a transparent plastic sealed cell (0.13 L) and a UV light source (ZF-1 three UV analyzer, Shanghai Jihui scientific analysis instrument Co., Ltd., China) (25 W, 365 nm). A piece of PVDF/meso-TiO_2_ membrane or PVDF/meso-TiO_2_/P123 membrane was added into the cell containing 20 mL of MO solution with different concentrations. In this study, two different ways were used to evaluate the photocatalytic degradation of MO. One was that the sample cell was kept in the dark without stirring for 6 h so that that the adsorption of MO by hybrid membrane reached equilibrium prior to the UV-irradiation. The other was that the sample cell was directly exposed to the UV-irradiation without stirring. Sampling was carried out at stated time intervals and degradation process of MO was monitored by a Cary 100 UV–Vis spectrophotometer (Varian Inc., USA). Concentration of MO was determined by its maximum absorption wavelength. Measuring method of hydroxyl radicals were similar to the previous report^46^ and described briefly as follows: a piece of PVDF/meso-TiO_2_ membrane was added into 20 mL of 10^−3^ mol L^−1^ COU aqueous solution in a dish with diameter of about 10 cm. A 350 W Xenon arc lamp (Shanghai Jihui scientific analysis instrument Co., Ltd., China) was used as a light source. PL spectra of generated 7HC were measured by a Varian Cary Eclipse fluorescence spectrophotometer (Varian Inc., USA). The excitation wavelength was 332 nm. During the Xenon lamp irradiation within 90 mins, the PL spectral of solution was recorded very 15 min. As for the adsorption experiment of 7HC by PVDF/meso-TiO_2_ membrane, a piece of PVDF/meso-TiO_2_ membrane was added into 10 μmol L^−1^ 7HC in a 10^−3^ mol L^−1^ COU aqueous solution, and the solution was detected by the same fluorescence spectrophotometer to measure the PL intensity at 456 nm very 15 min.

## Additional Information

**How to cite this article**: Wang, M. *et al.* Highly hydrophilic poly(vinylidene fluoride)/meso-titania hybrid mesoporous membrane for photocatalytic membrane reactor in water. *Sci. Rep.* **6**, 19148; doi: 10.1038/srep19148 (2016).

## Supplementary Material

Supplementary Information
