# Supplementary material for: Highly hydrophilic poly(vinylidene fluoride)/meso-titania hybrid mesoporous membrane for photocatalytic membrane reactor in water
Source: Sci Rep. 2016 Jan 12;6:19148. doi: 10.1038/srep19148 (PMC4709575; doi:10.1038/srep19148)
Supplement: Supplementary Information [file srep19148-s1.doc]

Supporting Information

**Highly hydrophilic poly(vinylidene fluoride)/meso-titania hybrid mesoporous membrane for photocatalytic membrane reactor**

*Meng Wang, Guang Yang, Peng Jin, Hao Tang, Huanhuan Wang & Yong Chen**


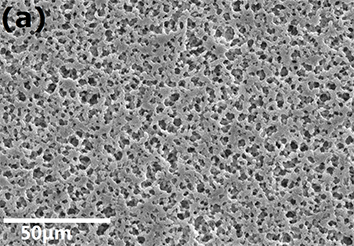

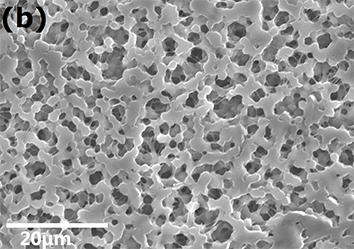

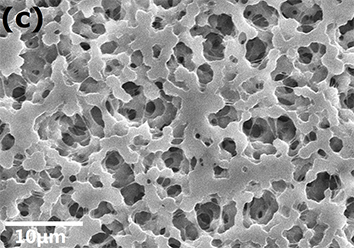

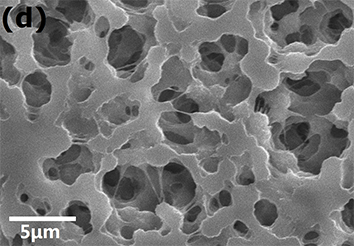


**Figure S1.** SEM images of the top-view of commercial PVDF membranes obtained under different magnification (a-d).


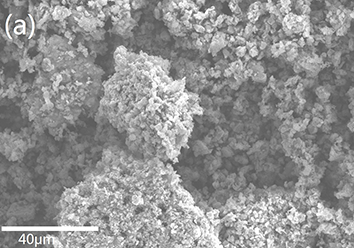

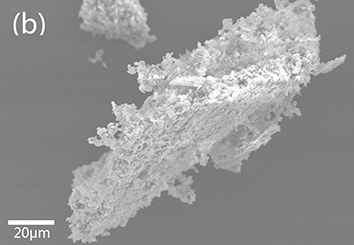

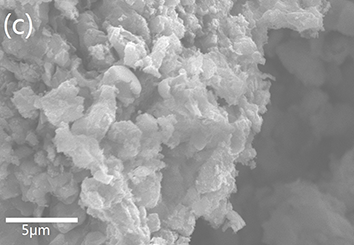

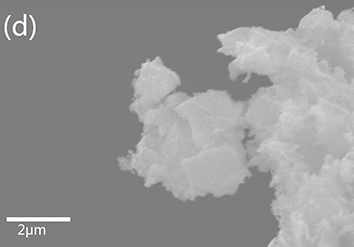


**Figure S2.** SEM images of the free layers released from PVDF/meso-TiO_2_ membrane under different magnification (a-d).


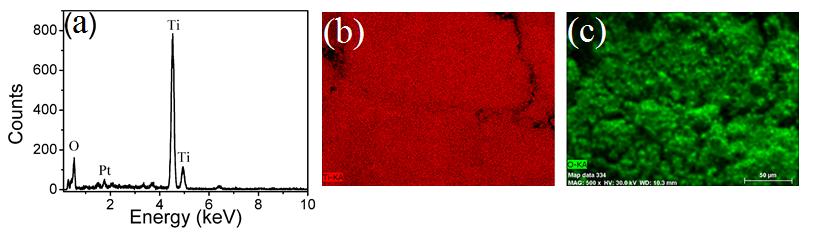


**Figure S3.** (a) EDS datum of the commercial PVDF membrane. EDS mapping of (b) titanium and (c) oxygen element of the free layers composed of rod-like materials released from PVDF/meso-TiO_2_.


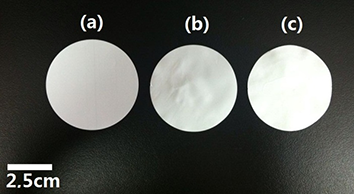


**Figure S4.** Photographs of commercial (a) PVDF, (b) PVDF/meso-TiO_2_ and (c) PVDF/meso-TiO_2_/P123 membranes.


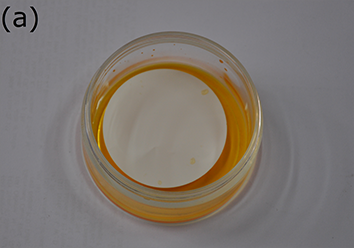




**Figure S5.** (a) Photograph of MO solution containing a commercial PVDF membrane; (b) the change of UV-vis absorption spectra of MO solution containing commercial PVDF membrane without (blue line) and with the irradiation of UV after 6 hours (red line).

| C_MO_  T | 1mg/L | 5mg/L | 10mg/L | 15mg/L | 20mg/L |
| --- | --- | --- | --- | --- | --- |
| 0h | 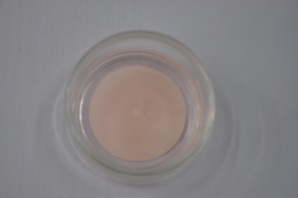 | 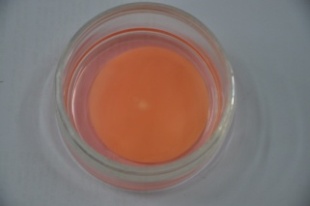 | 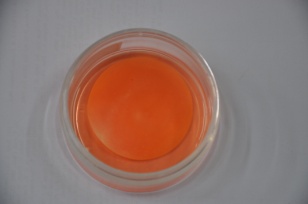 | 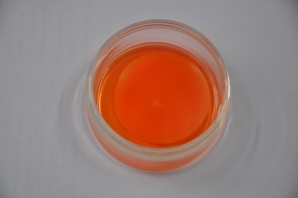 | 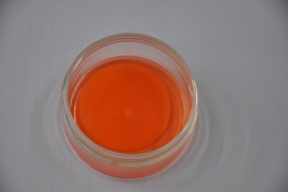 |
| 1h | 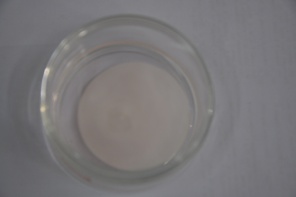 | 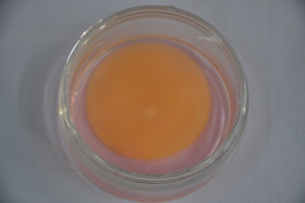 | 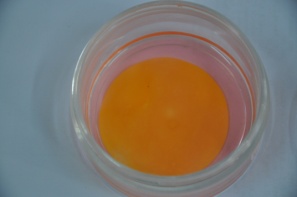 | 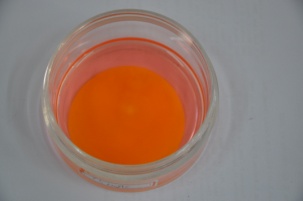 | 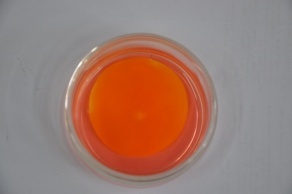 |
| 2.5h | 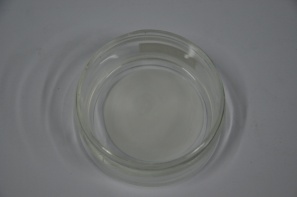 | 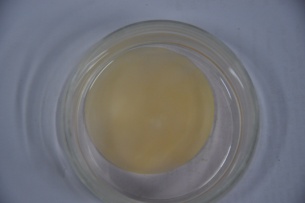 | 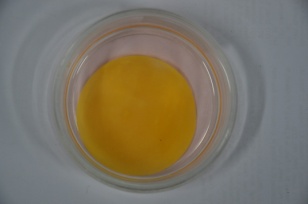 | 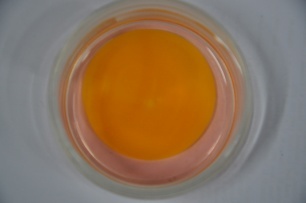 | 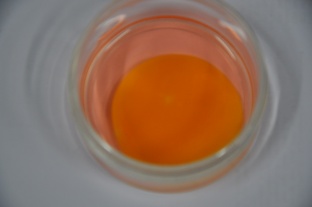 |
| 4h | 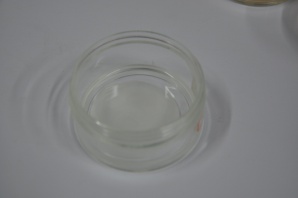 | 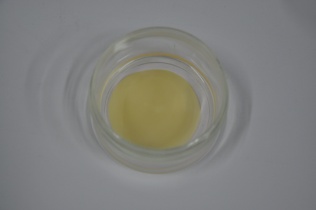 | 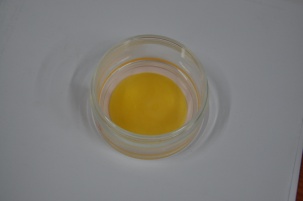 | 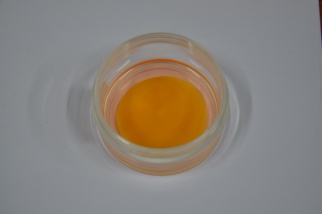 | 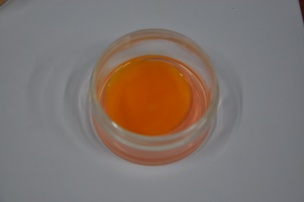 |
| 6h | 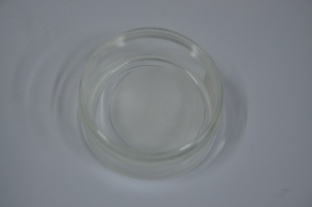 | 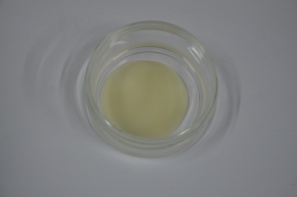 | 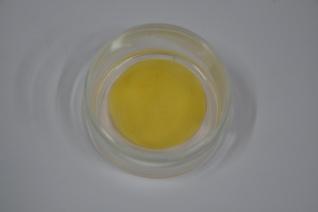 | 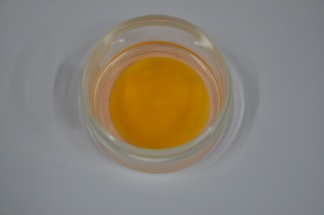 | 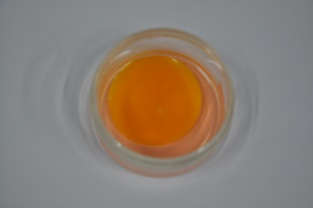 |
| 17h | 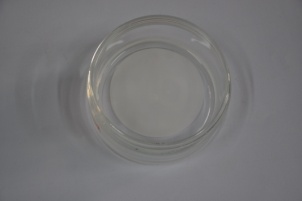 | 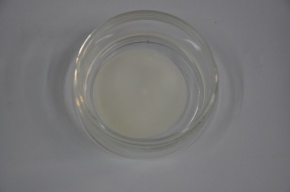 | 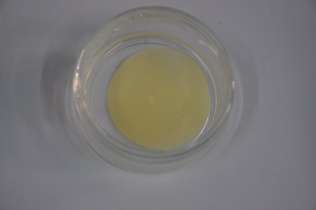 | 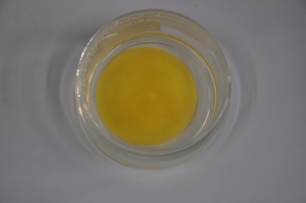 | 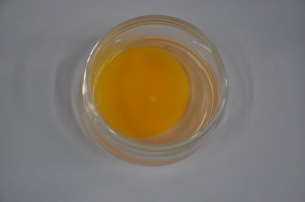 |
| 21.5h | 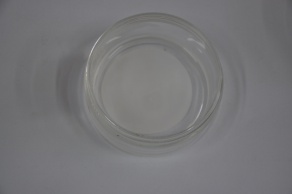 | 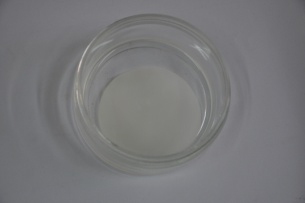 | 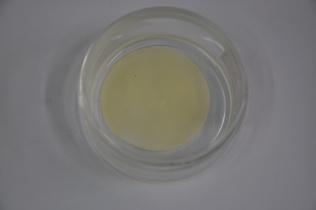 | 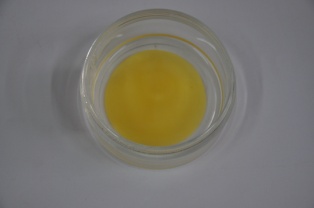 | 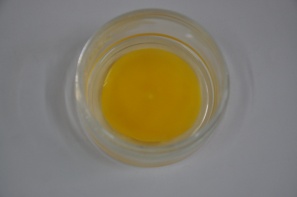 |
| 27h | 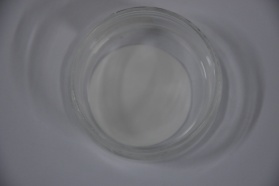 | 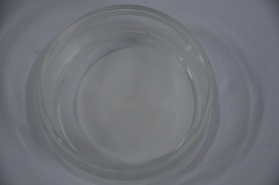 | 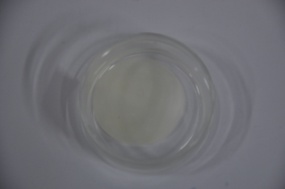 | 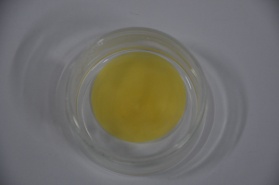 | 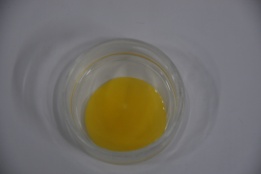 |

**Figure S6.** Photographs obtained for the photodegradation of MO with different concentrations (C_MO_) by using PVDF/meso-TiO_2_ membrane at the different time (T).


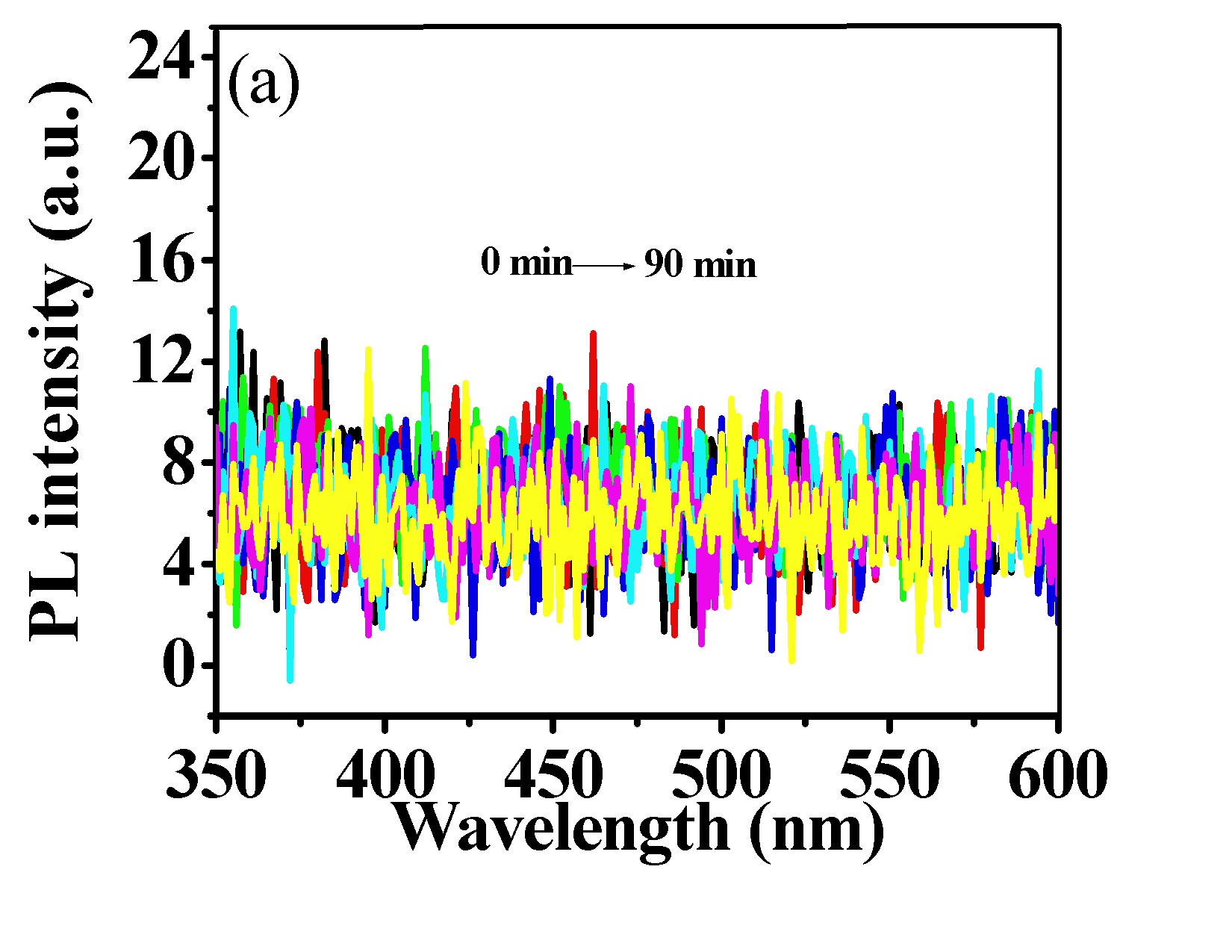

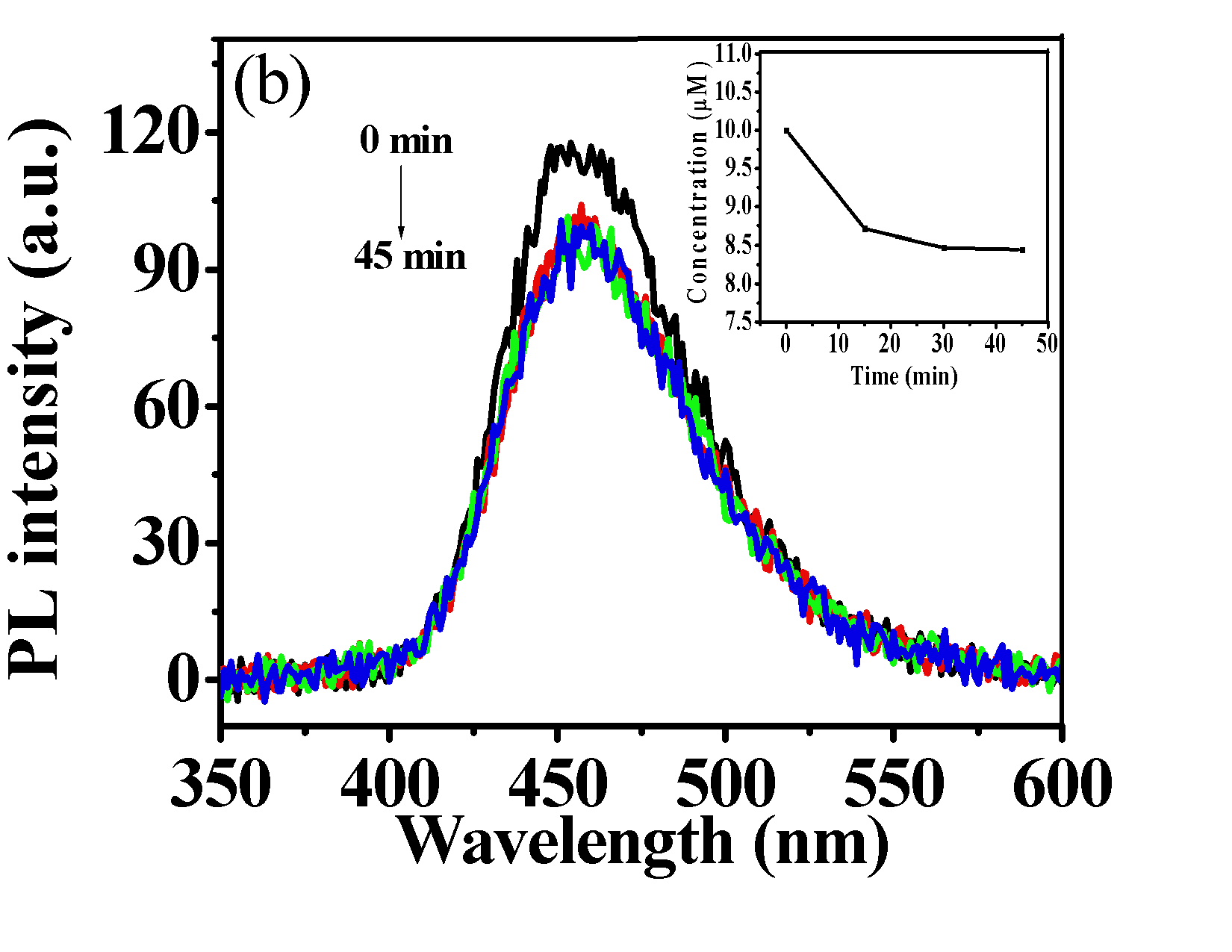


**Figure S7.**  (a) PL spectral changes observed during illumination of a piece of PVDF/meso-TiO_2_ membrane in a 10^-3^ mol L^−1^ COU aqueous solution. (b) PL spectral changes observed during illumination of a piece of PVDF/meso-TiO_2_ membrane in 10 μmol L^−1^ 7HC in a 10^-3^ mol L^−1^ COU aqueous solutiont. Inset in (b) shows the plot of concentration of 7HC against irradiation time. Each fluorescence spectrum was recorded very 15 min.


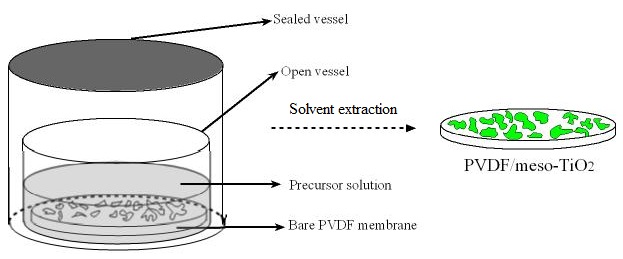


**Figure S8.** Scheme for the preparation process of PVDF/meso-TiO_2_ membrane.
